# Alkene selenenylation: A comprehensive analysis of relative reactivities, stereochemistry and asymmetric induction, and their comparisons with sulfenylation

**DOI:** 10.3762/bjoc.7.85

**Published:** 2011-06-03

**Authors:** Vadim A Soloshonok, Donna J Nelson

**Affiliations:** 1 University of the Basque Country UPV/EHU, San Sebastian, Spain; 2 IKERBASQUE, Basque Foundation for Science, 48011, Bilbao, Spain; 3 Department of Chemistry and Biochemistry, University of Oklahoma, Norman, OK 73019; 4 Department of Chemical Engineering, Massachusetts Institute of Technology, Cambridge, MA 02139

**Keywords:** alkene selenenylation, asymmetric synthesis, calculations, electronic effects, regioselectivity, relative reactivities, steric effects

## Abstract

A broad perspective of various factors influencing alkene selenenylation has been developed by concurrent detailed analysis of key experimental and theoretical data, such as asymmetric induction, stereochemistry, relative reactivities, and comparison with that of alkene sulfenylation. Alkyl group branching α to the double bond was shown to have the greatest effect on alkene reactivity and the stereochemical outcome of corresponding addition reactions. This is in sharp contrast with other additions to alkenes, which depend more on the degree of substitution on C=C or upon substituent electronic effects. Electronic and steric effects influencing asymmetric induction, stereochemistry, regiochemistry, and relative reactivities in the addition of PhSeOTf to alkenes are compared and contrasted with those of PhSCl.

## Introduction

Electrophilic addition to alkenes is one of the most fundamental, generalized, and versatile methods for selective functionalization of hydrocarbons [1]. Despite recent progress [2–3] in both synthetic and theoretical investigations of electrophilic selenenylation, it still remains one of the least studied types of electrophilic addition to alkenes, in particular when compared with sulfenylation. On the other hand, electrophilic selenenylation provides for the most straightforward and general methods for the preparation of the corresponding selenium derivatives, which are useful intermediates in organic synthesis, often with interesting biological applications [4–5]. Thus, organoselenium compounds are reported to have antitumor [6–8], antimicrobial [7–8], antiviral [7–8], and anti-oxidant [9–10] properties. It is interesting to note that the current applications of organoselenium derivatives have outpaced those of conventional inorganic selenium compounds [6–10]. This potential in biological applications [11] of organoselenium compounds is enhanced by their relatively low toxicity [8,12–14].

**Figure 1:**
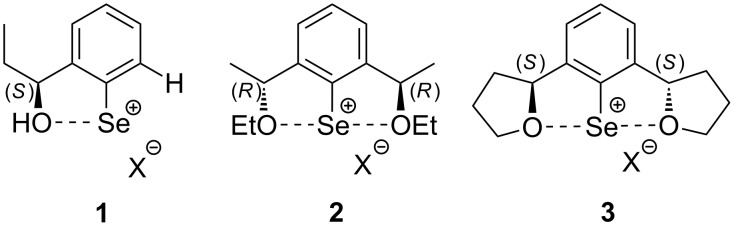
Chiral aryl selenium electrophiles **1**–**3**.

Of particular experimental and theoretical (computational) interest is the area of asymmetric selenenylation [15–43]. Drawing from the versatile reactivity of organoselenium compounds, it is expected that chiral selenium containing derivatives could be synthetically useful as chiral auxiliaries or intermediates for the development of a novel asymmetric methodology platform [15–18]. Furthermore, compared with sulfur analogs [44], chiral organoselenium compounds might be more powerful models for a systematic study of the self-disproportionation of enantiomers [45] via achiral chromatography [46–49] and sublimation [50–54]. One of the most developed approaches in this area is the application of chiral aryl selenium electrophiles of types **1**–**3** (Figure 1, usually X = OTf), containing a chiral alcohol/ether moiety *ortho* to the selenium atom [27–43]. Highly reactive electrophiles **1**–**3** are generated in situ from the corresponding diselenides via reaction with bromine followed by treatment with AgOTf [27–43]. While the stereochemical outcome of asymmetric selenenylations using compounds **1**–**3** heavily depends upon the reaction conditions and structure of a given alkene, *C*_2_-symmetric derivatives **2** and **3** are considered generally more efficient chiral auxiliaries compared with compound **1** [27–43].

**Scheme 1:**
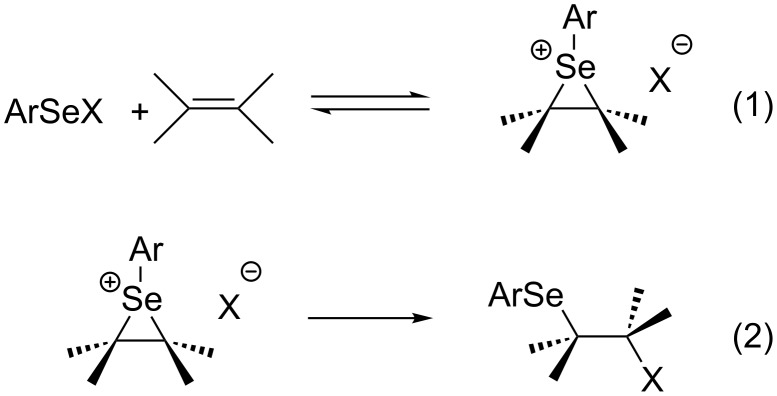
Plausible mechanism of alkene selenenylation.

Reactions of benzeneselenenyl halides, including chiral compounds **1**–**3**, with alkenes generally [55–56] exhibit high Markovnikov regioselectivity [57], with *anti* stereospecificity [58–59]. The nucleophile attacks the more substituted carbon in the seleniranium ion (Scheme 1), unless that carbon bears bulky groups, such as *tert*-butyl or cyclohexyl [55–56] or unless the open carbocation is stabilized, e.g., by an aryl group or a heteroatom [60–64].

The mechanism of electrophilic additions of ArSeHal to alkenes [65] (Scheme 1; X = Cl [60–64], Br [66]), in particular with ArSeCl, have been studied for some considerable time [67–68]. However, due to rather inconclusive results, these reactions are still of current theoretical and synthetic interest [69–70]. Thus, the step producing the seleniranium ion (reaction 1, Ar = Ph) [55–56,60–64,66], followed by *anti* attack by either an external or internal nucleophile [15–18,27–43,55–56,60–64,66,69–71], was initially reported to be rate-determining, but this step was later found to be reversible [17,34,69,71]. Therefore, there is remarkable disagreement in published rationales over which step determines the stereochemistry of the corresponding addition product:

1. The first step is suggested [37], because subsequent attack by the nucleophile is always *anti*;
2. the second step is suggested [34], because the first step is reversible;
3. and the first step for *trans*-alkenes, and the second step for *cis*-alkenes is also suggested [19].

There are also different theories on the role of a small amount of MeOH added to the reaction, including stabilization of the intermediate seleniranium ion [41] or its ion pair [64], as well as enabling a different reactive species, such as ArSeOMe [29]. The mechanism of these addition reactions is further complicated by noticeable influences such as reaction temperature [41,69] and the nature of the counter ion X [33,69]. Furthermore, additional factors stabilizing the seleniranium ion intermediate in step 1, including solvent effects [33,64], strengthening the Se–C bonds [34,55–56], or the presence of heteroatoms or aryl groups in close proximity to Se, were demonstrated to influence the mechanism by resonance stabilization [63] or complexation [31,39].

Relative reactivities of various alkenes toward the addition of PhSeCl [55] have been reported, and the importance of steric effects in the reaction has been emphasized [27–43,55–56,60–64,66]. However, the proposed [27–43,55–56,60–64,66] steric influences of the substituents on the C=C on the stereochemical outcome of these reactions have not been completely understood [29,33]. A more detailed analysis of steric and electronic effects might provide helpful mechanistic insights to clarify this practically important issue.

Previously, we demonstrated that additions to alkenes, which proceed through cyclic 3-membered intermediates usually of rate-determining transition states, exhibit characteristic patterns in their plots of log *k*_rel_ versus ionization energy (IE) or highest occupied molecular orbital (HOMO) energy [72–76]. These patterns were shown to be particularly useful to reveal the steric forces within, and electronic characteristics of, the rate determining transition states. Thus, plotting log *k*_rel_ values against IEs or HOMO energies of alkenes allows a comparison of the significance of steric and electronic effects in the rate-determining step of the reaction. In particular, additions to alkenes, that proceed via 3-membered intermediates (or transition states), display plots with one trend line if the reaction rate is predominantly dependent upon electronic effects [72–76], and multiple trend lines if both steric and electronic effects are important [72–73,77–78]. In this work, we apply these established relationships for a detailed analysis of the steric and electronic effects in the addition of PhSeCl to a series of representative alkenes and compare these findings with the corresponding data reported for the analogous sulfenylation reactions. Of particular interest and importance is the application of this approach for a more detailed and advanced understanding of the nature of the stereochemical outcome of the asymmetric selenenylation.

## Results and Discussion

### Building plots of log *k*_rel_ values for PhSeCl addition to alkenes versus their corresponding IEs and HOMOs values

Relative rates [55] of PhSeCl addition to representative alkenes in methylene chloride at 25 °C, alkene first ionization energies (IE) [79–80], and alkene highest occupied molecular orbital (HOMO) energies are compiled in Table 1. Only acyclic, unfunctionalized alkenes without aromatic substituents directly bonded to the C=C are included in this study to avoid undesirable complicating effects associated with ring strain, polarization, or conjugation [72–78]. Experimental IEs for alkenes in Table 1 are used as reported in the literature [79]. Alkene ab initio (HF level, 6-31G* basis set) HOMO energy values were calculated [81–83] and used in this study (Table 1). The particular values used were chosen, after comparing calculations by a variety of computational methods (Figures S1–S14 and Tables S1 and S2 in Supporting Information File 1), as these correlated best with alkene IEs and required reasonable computation time. Although this computational method may not give absolute HOMO energy values, it was successfully used in similar, previous studies [72–78], and it was proven to be sufficiently accurate in correlations with IEs and the relative rates of various addition reactions to alkenes. It should be noted that alkene HOMO energy calculations are particularly beneficial as the experimental IE data are usually incomplete or difficult to obtain. In particular, in the present study IE values for some di- and tri-substituted alkenes (Table 1) are, unfortunately, unknown, while these types of alkenes usually provide valuable mechanistic and stereochemical data.

**Table 1:** Representative alkene IEs (eV), HOMO energies (eV), relative rates (*k*_rel_), and log *k*_rel_ values of PhSeCl additions at 25 °C.

entry	alkene	IE^a^	HOMO	*k*_rel_^b^	log *k*_rel_

1	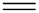	10.52	−10.18	11.6	1.06
2	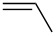	9.74	−9.72	100	2.00
3	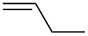	9.63	−9.70	76.1	1.88
4	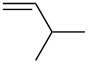	9.53	−9.69	11.7	1.07
5	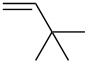	9.45	−9.65	7.4	0.87
6	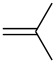	9.24	−9.39	77.2	1.89
7	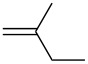	9.15	−9.37	25.3	1.40
8	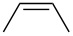	9.12	−9.28	42.8	1.63
9	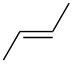	9.12	−9.29	23.8	1.38
10	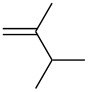	9.07	−9.36	10.6	1.03
11	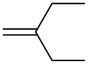	9.06	−9.34	14.1	1.15
12	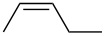	9.04	−9.27	94.4	1.97
13	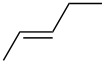	9.04	−9.28	42.5	1.63
14	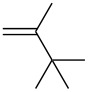	9.02	−9.36	3.6	0.56
15	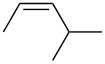	8.98	−9.27	17.3	1.24
16	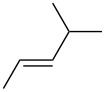	8.97	−9.28	14.0	1.16
17	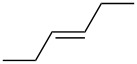	8.97	−9.27	31.9	1.50
18	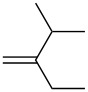	—	−9.32	2.35	0.37
19	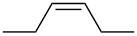	8.95	−9.27	59.9	1.78
20	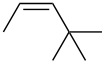	8.92	−9.27	20.9	1.32
21	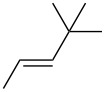	8.91	−9.28	0.31	−0.51
22	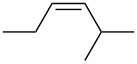	—	−9.27	14.71	1.17
23	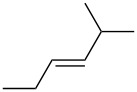	—	−9.27	8.61	0.94
24	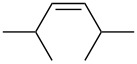	8.85	−9.27	0.41	−0.39
25	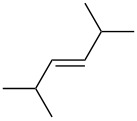	8.84	−9.27	0.35	−0.46
26	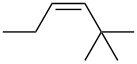	—	−9.24	20.0	1.30
27	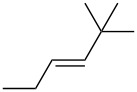	—	−9.27	0.10	−1.00
28	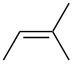	8.68	−8.99	43.0	1.63
29	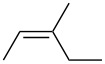	8.58^c^	−8.96	42.7	1.63
30	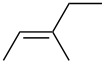	8.58^c^	−8.97	24.5	1.39
31	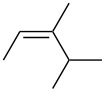	—	−8.96	6.39	0.81
32	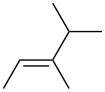	—	−8.96	2.05	0.31
33	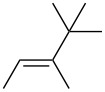	—	−8.95	0.01	−2.00
34	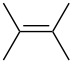	8.27	−8.73	28.1	1.45
35	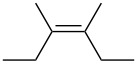	8.17	−8.68	0.96	−0.02
36	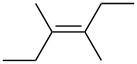	8.16	−8.65	0.50	−0.30

^a^Ref. [79], unless otherwise noted. ^b^Ref. [55]. ^c^Ref. [80].

**Figure 2:**
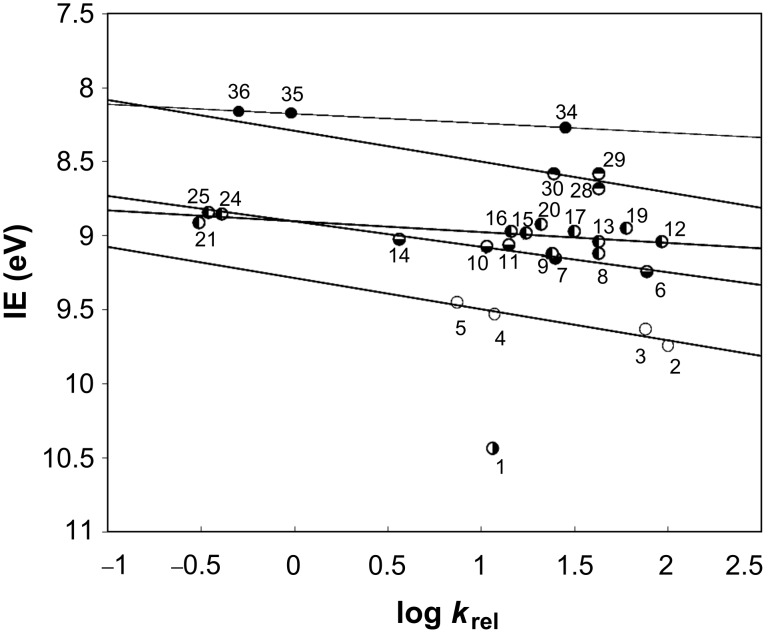
Plot of log *k*_rel_ values for PhSeCl addition to alkenes versus their corresponding IEs. Point numbers correspond to entries in Table 1.

In Figure 2, the plot of log *k*_rel_ values for PhSeCl addition to alkenes versus their corresponding IEs reveals a natural grouping into unsubstituted, mono-, di-, tri-, and tetrasubstituted alkenes. For each group of alkenes, as well as for all alkenes combined, the line of best fit is shown, and the correlation coefficient (r) [84] is given in the legend. The *y*-axis IE data are plotted in inverse order so that data reflecting lower π-electron energies appear at the bottom of the plot, in order to facilitate comparison with the plot of HOMOs. Most lines show a good-to-excellent [84] correlation (r_mono_ = 0.95, r_gem_ = 0.96, r_vic_ = 0.74, r_tri_ = 0.50, r_tetra_ = 1.00) within each sterically similar group. The correlation obtained by considering data for all alkenes is r_all_ = 0.32, much lower than that of any individual alkene group. Within each group of alkenes, the relative reaction rates show at most a very small increase with increasing IE; in some cases an almost horizontal line with near-zero slope is observed. The near-zero slope is probably due to the rate constants being dependent upon both the first step, involving electrophilic attack on C=C, and the second step, which is nucleophilic attack on the bridged intermediate by Cl^−^. As we demonstrated before, in this type of plot, electrophilic reactions give positive slopes [72–73,77], and nucleophilic reactions show negative slopes [76,78]. The combination of the opposing effects in the two steps would be expected to lead to a net canceling-out effect in the plot, giving the near-horizontal line obtained in this work.

**Figure 3:**
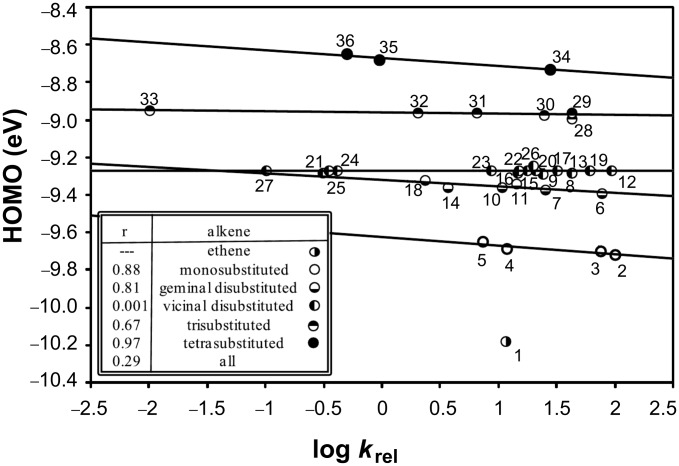
Plot of log *k*_rel_ values for PhSeCl addition to alkenes versus their corresponding HOMOs, analogous to the plot in Figure 2. Point numbers correspond to entries in Table 1.

For comparison, Figure 3 shows the analogous scatter of log *k*_rel_ values versus HOMOs of the corresponding alkenes (r_mono_ = 0.88, r_di_ = 0.15, r_gem_ = 0.81, r_vic_ = 0.001, r_tri_ = 0.67, r_tetra_ = 0.97 and r_all_ = 0.29). A natural grouping according to the number of substituents attached to C=C is again observed. Similar to the IE data, within each group of alkenes, relative reaction rates increase as the HOMO energies decrease.

### Analyzing steric effects in the addition of ArSeCl to alkenes

Detailed evaluation of the steric effects in the reactions under study requires careful consideration of the following factors: (A) Linearity within each group of points, (B) the significance of the multiple lines formed in Figures 1 and 2, and (C) different types of steric requirements of the alkyl groups attached to C=C.

#### Linearity of groups of points

One group of points, vicinal disubstituted alkenes, requires a special comment, as in both plots (Figure 2 and Figure 3) it appears to form a near-horizontal line. Usually, correlation coefficients are useful to determine how well groups of points form lines [84]. However, the unexpectedly low correlation coefficients, such as 0.001 (Figure 3), obtained in this work for some near-horizontal linear relationships are clearly at odds with their apparent linearity. Thus, according to theory [84–88], correlation coefficients calculated for relationships which have a slope of (or near) zero are undefined. For example, if the data form a perfectly horizontal line, the variance along the IE axis is zero, and the square root of this variance is in the denominator of the correlation coefficient equation [85–86]. Therefore, the observed discrepancy (low correlation coefficients) is due to the fact that this calculation method is not applicable for near-horizontal lines. To overcome this technical problem, it is necessary to use another approach, such as variance [84–88], to quantify how well the points in a given group fall on one line. Average variances for the data points, from their corresponding line fits in Figures 2 (IE) and 3 (HOMO), are given in Table 2.

**Table 2:** Average variance of points in each group based on their corresponding linear regression.

alkene group	variance (eV)	
	IE	HOMO

monosubstituted	0.01576	0.00087
geminal substituted	0.00767	0.00059
vicinal substituted	0.00839	0.00011
trisubstituted	0.00333	0.00019
tetrasubstituted	0.00370	0.00163
all	0.23326	0.09039

As seen in Table 2, variances for the vicinal disubstituted alkene lines are relatively similar in magnitude to those for monosubstituted, geminal disubstituted, trisubstituted, and tetrasubstituted alkenes. The obtained variance for each group is orders of magnitude less than that for all alkenes combined, which indicates that the linear fit is much better for groups of sterically similar alkenes than the group of all alkenes, as expected.

#### Significance of the separate line fits

The necessity for separate fitting of the sterically similar groups, seen in this study of selenenylation, is in perfect agreement with previously observed correlations (log *k*_rel_ versus IE or HOMO plots) reported for other types of addition reactions to alkenes [72–73,77]. On the other hand, it is remarkably different from the single line fit of positive slope, which has been obtained in analogous studies of arenesulfenylation [72]. The different line fits for sterically similar groups of alkenes, which are observed in the plots shown in Figure 2 and Figure 3, are obviously due to the dependence of IE on the degree of substitution at the C=C bond. The nearly horizontal lines formed by some groups of data points indicate that the steric effects on the reaction rates are not caused by a change in degree of substitution, but rather by a different type of steric effect.

#### Steric effects of alkyl substituents

The scatter plots for the reaction of alkenes with PhSeCl enable us to conduct a separate evaluation of three different sources of steric effects and to examine their relative influence on the reactivity and stereochemical outcome. These are:

1. The degree of substitution at the C=C bond (unsubstituted, monosubstituted, disubstituted, trisubstituted, or tetrasubstituted),
2. the relative positions of substituents at the double bond (vicinal or geminal), and
3. alkyl branching α to the C=C double bond.

1. Degree of substitution: The data points in Figure 2 and Figure 3 naturally cluster into five groups according to the number of alkyl substituents attached to the C=C bond. These five trend lines are almost parallel and only slightly separated from one another, indicating that increasing alkyl substitution on the double bond has a small overall effect. The small slopes of the trend lines also indicate that, within each group, there is no consistent relationship between relative rate and degree of substitution at the C=C bond. Thus, the data range widely in log *k*_rel_ values, so that no apparent relationship between log *k*_rel_ values and the number of substituents at the C=C bond is observed.

2. Relative positions of substituents: Relative rates depend on the relative positions of alkyl substituents attached to the double bond. For example, in disubstituted alkenes the alkyl substituents can be on the same carbon (geminal) or adjacent carbons (vicinal). Isobutylene reacts faster than *cis*- or *trans*-2-butene, but this is in the same order as their IEs and HOMOs, so it appears to be an electronic influence, rather than a steric effect. When the alkene has larger alkyl substituents, the general order of reactivity in PhSeHal addition is vicinal *cis*-alkenes > vicinal *trans*-alkenes > geminal alkenes, but the difference in reactivity between *cis* and *trans* is only about a factor of 2 or less (Table 1, entries 8 versus 9, or 12 versus 13). However, if one alkyl is *t*-butyl, then the difference is an order of magnitude greater (Table 1, entries 20 versus 21, or 26 versus 27).

3. Alkyl branching α to C=C: The data obtained in this study pointed to a new steric effect in linear free energy relationships, which can be defined as alkyl group α-branching. Thus, within each line fit, there is a clear trend dependent on steric–structural characteristics of the alkyl groups directly attached to the C=C bond. The data presented in Table 3 demonstrate this effect. Relative rates of the series of monosubstituted alkenes H_2_C=CHR decrease with increasing steric hindrance caused by branching at the α position; *k*_rel_ values for R = Me, Et, iPr, and *t*-Bu are 100, 76.1, 11.7, and 7.4, respectively. Geminal alkenes H_2_C=CMeR have smaller rates with a similar trend, which for R = Me, Et, iPr, and *t*-Bu correspond to 77.2, 25.3, 10.6, and 3.6, respectively. Trisubstituted alkenes of (*E*)-geometry HMeC=CMeR show an analogous trend, i.e., R = Me, Et, iPr, and *t*-Bu give *k*_rel_ values of 43.0, 24.5, 2.05, and 0.01, respectively. Replacing R = Me with R = *t*-Bu increases the rate-reduction by an amount which depends upon the steric congestion of the molecule (Table 3). In the relatively uncongested series H_2_C=CHR (Table 3, column 2), the rate is reduced by a factor of 13.5, from 100 to 7.4 (Table 1, entries 2 versus 5), but in the congested series HMeC=CMeR (Table 3, column 5) the rate is reduced by a factor of 4300, from 43.0 to 0.01 (Table 1, entries 28 versus 33). However, the progressive trend demonstrated in Table 3 is well-behaved only when the alkene series is unsymmetrical and/or sterically congested. For example, this trend is not observed in the vicinal disubstituted series HMeC=CHR. Therefore, the α-branching effect revealed in this study is different from what one might expect based on the usual general consideration of the total steric bulk of alkyl substituents.

**Table 3:** Effect of substituent branching upon PhSeCl addition in alkene systems.

R	relative rates (*k*_rel_)			

	H_2_C=CHR	H_2_C=CMeR	HEtC=CHR (*E*-isomer)	HMeC=CMeR (*E*-isomer)

Me	100	77.2	42.5	43.0
Et	76.1	25.3	31.9	24.5
iPr	11.7	10.6	8.61	2.05
*t*-Bu	7.4	3.6	0.10	0.01

**Figure 4:**
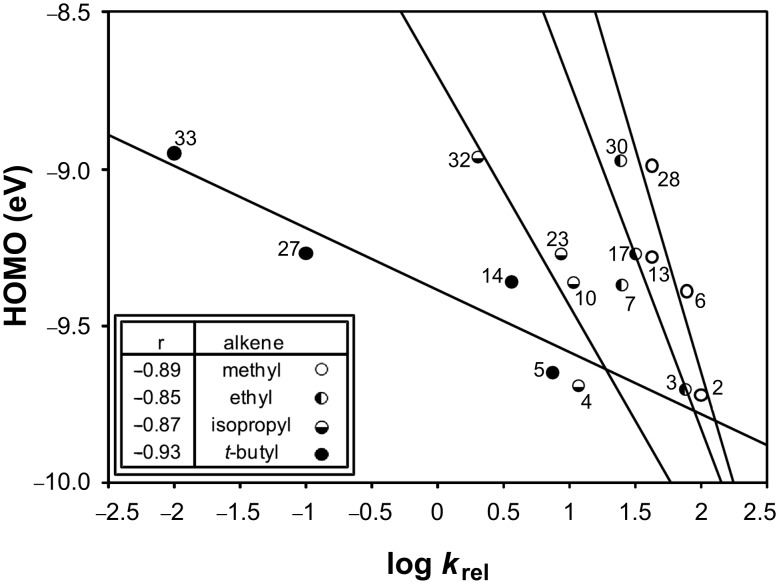
Plot of log *k*_rel_ versus HOMO shows data grouped by branching at α position. Data are from Table 3; point numbers correspond to entries in Table 1.

This α-branching effect is further demonstrated by the plot in Figure 4, in which the fastest reacting compound from each series is designated by hollow circles (○), and the corresponding slowest reacting derivative is designated by filled circles (●). These plotted relationships are reasonably linear, demonstrating consistency in rate reductions due to combined steric effects. The total steric effects are greater when R is geminal to another substituent, as expected.

The multiple fit lines and the pattern of alkenes within each line clearly indicate that overall steric effects are much more influential than electronic effects upon the rate of this reaction. However, of the three steric effects considered, branching of alkyl groups α to the double bond has the greatest and most consistent influence on the reaction rate.

### Stereochemical outcome of addition reactions of chiral ArSeOTf to alkenes

#### Stereoselectivity

**Scheme 2:**
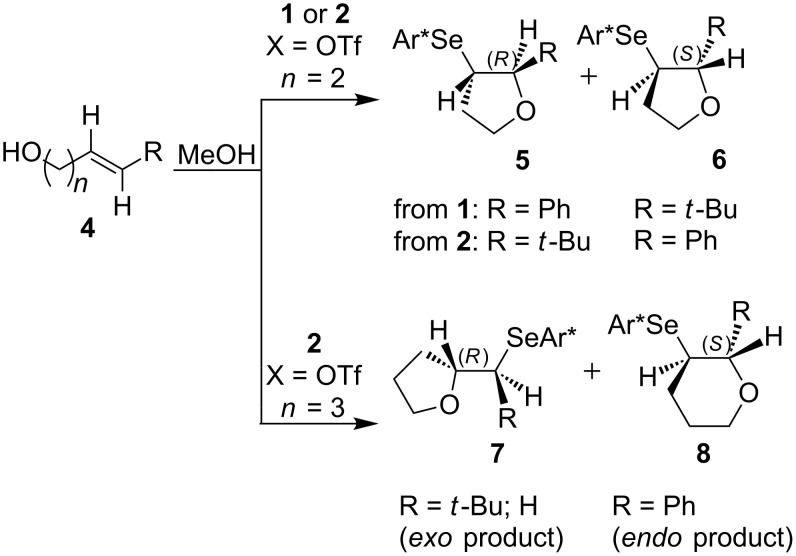
Major products from reactions of **1** and **2** with representative alkenols.

Some experimental and computational [27–43,89] data suggest that, in the reactions of alkenols such as **4** (Scheme 2) with the chiral selenium electrophile, the stereochemistry of the intermediate seleniranium ion can be efficiently controlled [71]. It is assumed that the first reversible [69] step of the reaction determines the absolute configuration of the products and the second, rate-determining step, controls the regiochemistry [15–18,27–43,60–64,66,69–71]. Scheme 2 shows the reactions of **1** and **2** with alkenols **4**, and the stereochemical outcome of each is compiled in Table 4. It should be noted that the absolute configuration of products from reactions of **1** and of **2** with alkenols **4** is reversed, because of the opposite stereochemistry of the chiral stereogenic centers in **1** versus **2**. While not included in this study, it should be emphasized that analogous stereochemical outcomes were observed in the corresponding reactions of chiral electrophiles **1** and **2** with carboxylic acids of general formula RCH=CH(CH_2_)*_n_*COOH (*n* = 1, 2) used instead of alkenols **4** [29].

**Table 4:** Characteristics of major products from reactions of **1** and **2** with alkenols in Scheme 2 at −78 °C.

	**1** (*n* = 2)				**2** (*n* = 2)				**2** (*n* = 3)			
R	de	R:S (dr %)	% yield	ref.	de	R:S (dr %)	% yield	ref.	de	R:S (dr %)	% yield	ref.

Ph	84	92:8	87	[41]	84	8:92	92	[29]	93	3.5:96.5	95	[29]
Me	0	50:50	—^a^	[33]	0	50:50	—^a^	[33]	0	50:50	—^a^	[33]
Et	0	50:50	60	[41]	34	67:33 or 33:67^b^	73	[29]	40^c,d^	70:30 or 30:70^b^	96^c,d^	[29]
*t*-Bu	46	27:73	68	[41]	84	92:8	77	[29]	80^d^	90:10	89^d^	[29]

^a^Not reported. ^b^Major diastereomer stereochemistry was not assigned. ^c^R = H. ^d^*exo* product [36,41] is obtained.

#### Effect of steric requirements on chiral ArSeOTf

It was demonstrated that branching in R on O in reagent **1** (Figure 1), such as replacing H with progressively larger alkyl groups, decreased the diastereoselectivity in the formation of corresponding addition products [43]. In contrast, chiral derivative **2**, possessing two ethoxy groups, provides for higher diastereoselectivity. While the mode of asymmetric induction in the reactions of compounds **1** and **2** is obviously different, the higher diastereoselectivity observed in the additions of **2** could not be fully explained by its *C*_2_-symmetric structure or by increased seleniranium intermediate stability via coordination to two oxygens in **2** [33]. Thus, compound **3**, also with a *C*_2_-symmetric structure, provides for even higher stereochemical outcome in the corresponding addition reactions. For example, in the reaction of **3** with (*E*)-6,6-dimethyl-4-hepten-1-ol, a product of type **7** (Scheme 2) was isolated with 87.5% de, while the analogous reaction using compound **2** gave product **7** in 80% de (R = *t*-Bu and *n* = 3 in Table 4) [32,34]. Since compound **3** has lower steric requirements than that of **2** (tethered alkyl groups as part of the ring instead of freely-rotating groups), these stereochemical results clearly support the trend (steric bulk versus diastereoselectivity) observed for compounds of type **1** [34,43]. Furthermore, it was shown that the stereochemical outcome of the asymmetric selenenylation is noticeably more dependent on the structure of the starting alkene rather than that of chiral compounds **1**–**3**. Considering these experimental observations and taking into account the importance of steric effects revealed in this work, one may conclude that the major source of stereochemical preferences should be within the structure of the alkene.

#### Effect of an Ar substituent on C=C

The presence of an Ar substituent directly bonded to the C=C bond of the starting alkene has a dramatic effect on the stereochemical outcome of the asymmetric selenenylation. For example, the reaction of **2** with 4-phenyl-3-buten-1-ol (Scheme 2, Table 4, *n* = 2) gives product **6** with 84% de in 92% yield. When R = Et, the diastereoselectivity and yield decrease to 34% and 73% respectively. The higher level of asymmetric induction and yield in the case of R = Ph has been attributed to a stabilizing π–π stacking of alkene and electrophile substituents [33–34]. Results for **1** and **2** (*n* = 3) in Table 4 also, in general, follow this pattern. In the reactions of **1** or **2** with alkenols **4** (*n* = 3) (Scheme 2), the stabilizing effects of phenyl at the developing positive charge at the adjacent carbon are considered to be a reason for the predominant *endo* cyclization furnishing products **8** [29] (Table 4).

#### Effect of alkyl substituents on C=C

Reaction of compound **2** with (*E*)-5,5-dimethyl-3-hexen-1-ol (**4**, R = *t*-Bu, *n* = 2) yields the corresponding product **5** with high diastereoselectivity (84% de) and with the opposite absolute configuration as compared to the phenyl containing starting alkene previously discussed. The stereochemical outcome decreases to 34% de when (*E*)-3-hexenol (R = Et, *n* = 2) is used [29,34,41].

The reduced reactivity observed for PhSeCl addition to (*E*)-5,5-dimethyl-3-hexene compared to trans-3-hexene (Table 1, entries 27 versus 17, a factor of ~320) would also be expected in this system, and a higher selectivity for R = *t*-Bu than R = Et would be expected to accompany the reduced reactivity. The observed higher selectivity for R = *t*-Bu supports asymmetric induction being influenced greatly by steric effects at the α position. Data in Table 4 for **1** and for **2** (*n* = 3) also follow this pattern. The diastereoselectivity of products from reactions of alkenes with R = *t*-Bu are reversed from those with R = Ph, because the interactions of the former with **1** or **2** are destabilizing, while those with the latter are stabilizing.

All of the alkenes that showed a high level of diastereoselectivity in this reaction are *trans* substituted [27–43], which was attributed to a reduced reactivity of *cis* alkenes [41]. However, it is also reported that *cis* alkenes generally react faster than *trans* derivatives [57] (see Table 1), and a lower reactivity of *trans* alkenes is a more reasonable explanation for their higher selectivity to produce greater asymmetric induction in this reaction.

#### Effect of branching α to the C=C; computational studies

**Figure 5:**
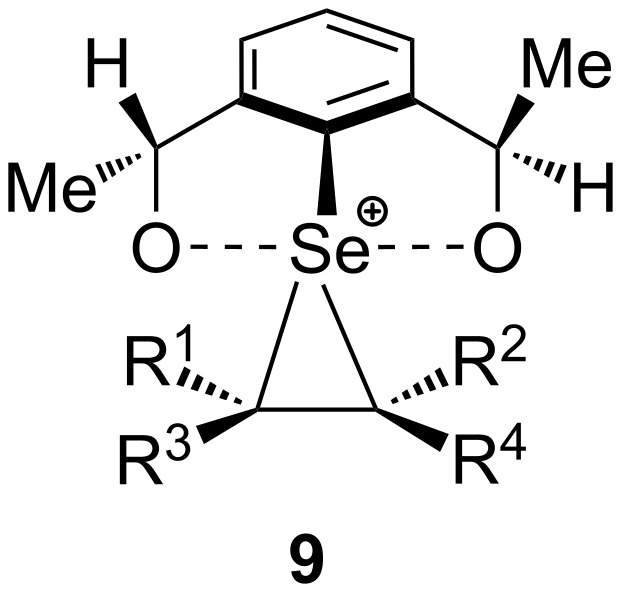
Structure of intermediate complex **9**.

The reaction of *C*_2_-symmetric electrophile **2** with olefins R^1^R^3^C=CR^2^R^4^ gives an intermediate with a structure similar to that of **9** (Figure 5) [32–39]. Computational studies [33–34] of complexes formed in reactions of **1** and **2** with alkenes were carried out by using B3LYP/6-31G*, in order to explain the stereochemistries and regiochemistries shown in Scheme 2 and Table 4. The energy of **9** was found to depend on, not only the substituent attached to C=C, but also its position in **9**, as shown in Table 5. The phenyl substituted isomer of **9**, with Ph at position R^2^ in order that it may interact more with Ar* on Se in a π – π-stacking stabilization [33–34], is reported to give the major product. This produces asymmetric induction favoring the (*S*)-isomer in the reaction of **2** with the analogous phenyl substituted alkenols 4 (R^2^ = Ph, R^3^ = –(CH_2_)*_n_*–OH, *n* = 2 or 3) [33–34], as shown in Scheme 2 and Table 4.

**Table 5:** Energy increase in **9** upon substitution at the α position [34].

alkyl groups and their positions				energies of isomers (kcal/mol)	

R^1^	R^2^	R^3^	R^4^	*E*_rel_	*E*_branching (_*_t_*_-Bu–Me)_

H	H	H	H	0	—

Me	H	H	H	2.3	—
H	Me	H	H	1.4	—
H	H	Me	H	0	—
H	H	H	Me	0	—

*t*-Bu	H	H	H	9.9	7.6
H	*t*-Bu	H	H	7.3	5.9
H	H	*t*-Bu	H	0.6	0.6
H	H	H	*t*-Bu	0	0

The effect of alkyl groups attached to the C=C bond on asymmetric induction was attributed to overall steric bulkiness [33–34], however, with our approach it is possible to analyze the steric effects more specifically. For example, little if any consistent change in the asymmetric induction is observed in Table 4, whether R = H, Me, or Et. However, branching α to C=C has an effect upon the asymmetric induction which is approximately equal to, but in the opposite direction of, that of Ph. This branching was also explored by using the computational data [34] in which Me is replaced with *t*-Bu, which increases the energy of complex **9**. The greatest energy increase (Me → *t*-Bu = 2.3 → 9.9 = 7.6 kcal/mol) occurs when the substituents are in position R^1^. At this position, the alkyl is closest to the Me of **9** resulting in strongly destabilizing repulsive steric interactions. Branching causes the smallest energy increase (Me → *t*-Bu = 0 → 0 = 0 kcal/mol increase) at position R^4^. Therefore, complex **9** is most stable with the *t*-Bu group occupying this sterically most favorable position. In Table 4, the diastereoselectivity increase as a result of increased branching α to the C=C bond indicates increased chiral recognition and consequently, asymmetric induction. As shown in Table 5, this is caused by increases in energy upon branching α to the C=C (at R^1^, R^2^, and R^3^). The higher energies of the corresponding intermediates and transition states produce greater selectivity for the R^4^ = *t*-Bu isomer of **9**, and therefore greater selectivity in transfer of chiral information.

The findings discussed above, indicating that major steric effects are incurred at the α position, agree (1) with reports that asymmetric induction is increased by substitution at that position and (2) with results showing that the major steric effects in the relative rates of PhSeCl addition to alkenes are caused by branching α to C=C, as reported herein. It was found that MeOH was necessary [64] to achieve high asymmetric induction and yields, but MeOH was not used in the study [57] where the relative reactivities shown in Table 1 were determined. This effect of branching also seems to hold regardless of differences in the counter ion (Cl^−^ versus OTf^−^) and temperature (25 °C versus −78 °C). Thus, similar conclusions about the significance and type of steric effects have been reached for these different systems, which indicates that the steric recognitions are inherent interactions between the reactants themselves and not noticeably influenced by other factors (e.g., reaction conditions).

#### Comparing characteristics of ArSeX versus ArSCl addition to alkenes

Similarities between the reactions of sulfur and selenium compounds might be expected, because sulfur and selenium belong to the same group in the periodic table, and it has been proposed that both reactions could follow similar mechanistic pathways [57]. Indeed, arenesulfenyl and areneselenenyl chlorides react with alkenes to yield the corresponding β-chloroalkyl aryl sulfides and selenides, respectively. Conversely, differences between the two reactions have been reported, but the sources of these differences have not been fully explained:

1. While arenesulfenyl chlorides add to alkenes with an anti-Markovnikov orientation, areneselenenyl chlorides add with Markovnikov orientation [34,55,57,63,69]. However, the orientation can be significantly influenced, or even reversed, by the steric bulk of substituents in the alkene [34,55], by changing the counterion [66,89], by aryl substituents on the C=C [19,60], or by added solvents such as methanol [27,64,89].
2. It is well established that the rate determining step in benzenesulfenyl chloride addition is the formation of thiiranium intermediate; specifically the alkene π electrons displace Cl^−^ in an S_N_2 reaction to give the thiiranium ion [89]. On the other hand, recent studies ruled out the formation of the corresponding seleniranium ion as the rate-determining step in PhSeCl addition under the reaction conditions used to obtain the data analyzed herein [17,34,69,71].
3. A non-cumulative effect of methyl substituents upon the rate of addition of PhSeCl to alkenes [57] was contrasted against a cumulative effect of methyl substituents upon the rates of reaction in several other electrophilic additions to alkenes, including arenesulfenyl chlorides. This is due to steric effects predominating in the former, while electronic effects predominate in the latter.
4. Stereochemical outcomes in the asymmetric reactions of chiral arenesulfenyl chlorides [91] generally feature both lower diastereoselectivity and chemical yields as compared with analogous asymmetric reactions of areneselenenyl chlorides. Each of these differences is in agreement with the findings discussed in this work, that increasing the degree of substitution at the C=C bond has different effects on *k*_rel_ values of the two reactions.

#### Differences in rate determining steps

**Figure 6:**
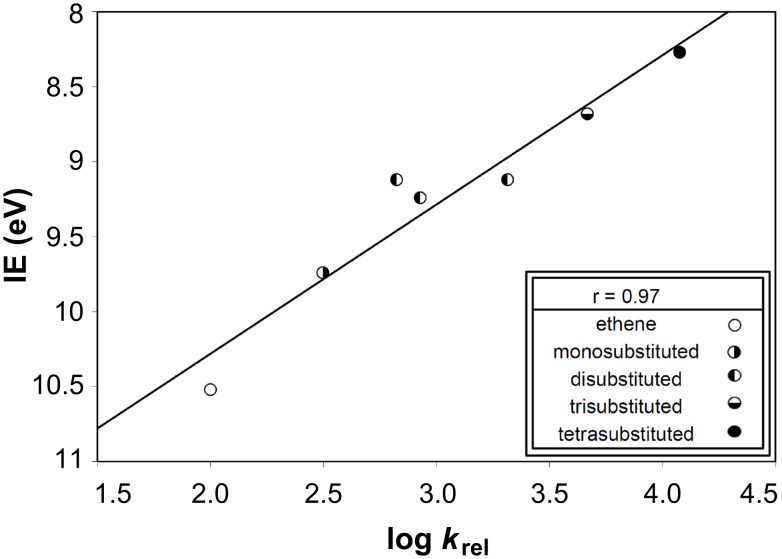
Plot of log *k*_rel_ values for PhSCl addition to alkenes versus their IEs. Data are from Table 6.

Differences in the reported rate-determining steps for the additions of PhSeCl and PhSCl to alkenes prompted a comparison of their plots of log *k*_rel_ values versus alkene IEs. The plot for the former is shown in Figure 1. The plot for the latter, using data given in Table 6, is shown in Figure 6. Different data point groupings are obtained in these plots for the two reactions; the scatter plot for areneselenenyl chloride addition to alkenes gives multiple lines (Figure 1), while arylsulfenyl chloride addition [89] gives a single trend line, both herein (Table 6, Figure 6, r_all_ = 0.97) and previously [72] with a different set of relative rate data. Studies of other additions to alkenes which proceed through 3-membered rate-determining stationary points [72–78] have demonstrated that such different data point groupings reflect different steric and electronic characteristics of the rate-determining transition states, intermediates, or products. These different patterns have corresponded to different reaction mechanisms [72–78]. Thus, the different changes in alkene reactivities between the two reactions reflect different changes in structure between the two reactions.

**Table 6:** Alkene IEs (eV), relative rates (*k*_rel_), and log *k*_rel_ values of PhSCl addition to olefins.

alkene	IE^a^	*k*_rel_^b^	log *k*_rel_

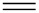	10.52	100	2.00
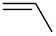	9.74	314	2.50
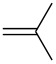	9.24	846	2.93
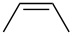	9.12	2060	3.31
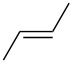	9.12	666	2.82
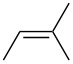	8.68	4650	3.67
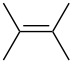	8.27	11900	4.08

correlation coefficient (r)			0.97

^a^Ref. [29]. ^b^Ref. [35]

The plot showing a single line trend for PhSCl addition to the C=C bond supports the idea that the reaction rate is predominantly dependent upon electronic effects, with steric effects being relatively unimportant, as previously observed [72–77]. The plots with multiple trend lines for PhSeCl addition to the C=C bond indicate that steric effects are significantly more important [72–73,77–78]. For reactions each involving a bridged, 3-membered cyclic stationary point, a plot with a single trend line indicates that steric effects are of low importance and points to a rate-determining transition state preceding the formation of a cyclic intermediate. Conversely, a plot with multiple trend lines indicates a greater importance of steric effects and points to a rate-determining transition state following the formation of 3-membered cyclic intermediate. In the case of PhSeCl, the steric effects are primarily due to the degree of branching α to the C=C bond, and then, to a lesser extent, to the number of substituents attached to the C=C bond. In the previously-reported reactions displaying multiple lines, the steric effects were due to the number of substituents attached to the C=C bond.

These correlations agree with previous mechanistic investigations in the PhSeCl and PhSCl additions to alkenes. The rate determining step in benzenesulfenyl chloride addition is reported [89–92] to be thiiranium intermediate formation, specifically via electrophilic attack by PhSCl upon the alkene [90]. On the other hand, as noted above, recent studies rule out formation of the corresponding seleniranium ion as the rate-determining step in PhSeCl addition [17,34,69,71], stating that the first step is reversible and that the rate determining step follows the seleniranium ion intermediate in the mechanism. Both of these fit the patterns established in this work as observed in plots of log *k*_rel_ versus IE or HOMO for additions to alkenes.

#### Effect of alkene methyl substituents upon reaction rate

As discussed above, the data and plot for areneselenenyl chloride addition to alkenes indicates significant steric effects, while the arylsulfenyl chloride addition data (Table 6) and plot [72] (Figure 4) indicate a relative independence from steric effects and a predominant influence of electronic effects on the reaction rate. These observations agree with previous studies on the effect of increasing the number of methyl substituents on the C=C bond. A non-cumulative (rate decreasing) effect of methyl substituents upon the rate of addition of PhSeCl to alkenes [57] was previously contrasted against a cumulative acceleration by methyl substituents upon the rate of reaction with ArSCl; the substituent effect comparison was not extended beyond methyl, so this has no bearing on the effects of branching. Nevertheless, this difference agrees with the different number of trend lines in the ArSeCl addition plot (Figure 1) versus the ArSCl addition plot (Figure 6) [72,89].

#### Chiral induction and steric effects in arenesulfenyl (ArSCl) and areneselenenyl (ArSeOTf) additions to alkenes

There are far fewer reports of chirality induced by arenesulfenyl chlorides [91], which are analogous to areneselenenyl compounds **1** [32–43]. Data in Table 7 compare electrophilic cyclizations of alkenes by using compound **1** (X = OTf) [32–43,91] and compound **10**, which is the sulfur analog of the chiral selenium compound **1** (X = Cl) [80]. Both **1** and **10** react to give products with the same absolute configuration. Furthermore, both reactions undergo *endo* or *exo* cyclization as necessary in order to produce a 5-membered ring in the product. These data reveal that asymmetric induction [80] by the chiral arenesulfenyl chloride **10** gives lower yields and generally lower diastereoselectivity compared with the analogous areneselenenyl chlorides **1**, especially when there are higher steric requirements in the alkene. As discussed above, this lower selectivity also supports steric effects being less important in ArSCl addition to alkenes than in ArSeCl addition.

**Table 7:** Electrophilic cyclizations of alkenes with electrophiles **1** (X = OTf) and **10**.

reaction		yield / dr (R:S)	
alkene	product	**1** (X = OTf)	**10** [35]

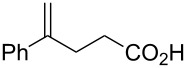	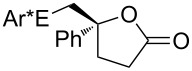	58% / 92:8 [41]	38% / 89:11
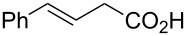	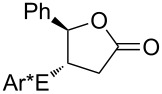	41% / 86:14 [41]	5% / 70:30
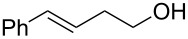	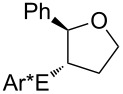	87% / 92:8 [41]	4% / —
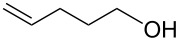	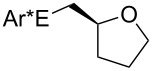	60% / 50:50 [90]	10% / 65:35

## Conclusion

Multiple methods demonstrate herein the importance of the effects of branching α to the C=C bond upon ArSeX addition to alkenes.

1. The relative importance of different steric effects of alkene substituents, which influence reactivities of alkenes toward PhSeCl, were analyzed by a simple method. Plots of log *k*_rel_ versus IEs and versus HOMO energies reveal multiple nearly-parallel lines of best fit with small slopes in each. Thus, due to their relatively small slopes, these multiple trend lines indicate that IEs and HOMO energies are dependent upon increasing substitution at the C=C bond, in a different manner to other additions which also displayed multiple trend lines in such plots [72–78]. Overall, the natural grouping into mono-, di-, tri-, and tetrasubstituted alkenes gave better correlation coefficients than that obtained for all alkenes, analogously to other additions which displayed multiple trend lines in such plots. The greatest effect on the rate of PhSeCl addition to alkenes was due to branching of alkyl groups α to C=C, rather than electronic effects or total steric bulk related to the degree of substitution on the C=C bond, relative positions of alkyls, or their sizes.
2. Branching of alkyls α to C=C was also found to be the most important effect responsible for asymmetric induction in the reaction under study.
3. The stereoselective and regioselective outcomes of selenenylation reactions, such as those using compounds **1** and **2**, were explained by interactions between reactants and within reaction intermediates, which are due to the steric requirements of substituents on both the alkene (R ≠ Ph) and the electrophile. Calculations of the reaction intermediate **4** with substituents on the C=C bond reveal a much higher energy with R = *t*-Bu than with R = Et. The lowest-energy conformation of **9** with greatest branching α to C=C (R = *t*-Bu) corresponds to the greatest experimentally observed asymmetric induction.
4. Although PhSCl and PhSeCl react with alkenes to give similar products, the reaction rate of the former depends mainly upon electronic effects, while the latter is influenced predominantly by steric effects. The mechanistic pathway of the latter, which leads from a cyclic three-membered structure in the rate determining step (Scheme 1), transfers these observed steric effects to the transition state (Scheme 1, Equation 2) [69–70,89]. However, the different number of trend lines in the plots of log *k*_rel_ values versus IEs for PhSeCl and PhSCl addition is consistent with different mechanisms for the two reactions. Thus, each plot of log *k*_rel_ values versus IEs for ArSCl has a single trend line [72,89] whilst that for ArSeCl (Figure 1) has multiple trend lines, in which alkenes are grouped according to their steric requirements.

## Experimental

Two tables and fourteen scatter plots of alkene IEs versus HOMO energies calculated by four different methods and of alkene EAs versus LUMO energies also calculated by four different methods are shown in the Supporting Information File 1. Four computational methods, including an ab initio method at HF level in five different basis sets (3-21G^(*)^, 6-31G*, 6-31+G*, 6-311G*, and 6-311+G*), two semi-empirical methods (PM3 and MNDO), and a DFT method (B3LYP/6-31G*), were used to obtain the calculated data [81–83]. The minimum energy geometries were calculated for each molecule, with full geometry optimization.

## Supporting Information

Supporting Information features detailed calcualtion data for IEs, HOMOs, LUMOs and related data.

File 1 Alkene IEs, HOMO energies, EAs, and LUMO energies, and related data.
