# Supplementary material for: Alkene selenenylation: A comprehensive analysis of relative reactivities, stereochemistry and asymmetric induction, and their comparisons with sulfenylation
Source: Beilstein J Org Chem. 2011 Jun 3;7:744–58. doi: 10.3762/bjoc.7.85 (PMC3135076; doi:10.3762/bjoc.7.85)
Supplement: File 1 — Alkene IEs, HOMO energies, EAs, and LUMO energies, and related data. [file Beilstein_J_Org_Chem-07-744-s001.pdf]

Supporting Information

for

Alkene selenenylation: A comprehensive analysis of relative reactivities, stereochemistry and asymmetric induction, and their comparisons with sulfenylation

Vadim A. Soloshonok[1,2] and Donna J. Nelson*[3,4,$]

Address: [1]University of the Basque Country UPV/EHU, San Sebastian, Spain; [2]IKERBASQUE, Basque Foundation for Science, 48011, Bilbao, Spain; [3]Department of Chemistry and Biochemistry, University of Oklahoma, Norman, OK 73019; and [4]Department of Chemical Engineering, Massachusetts Institute of Technology, Cambridge, MA 02139

Email: Vadim A. Soloshonok - v.soloshonok@ikerbasque.org; Donna J. Nelson - DJNelson@ou.edu

* Corresponding author

[$] alternative email address: djnelson@mit.edu

Alkene IEs, HOMO energies, EAs, and LUMO energies, and related data

Table S1: Correlation coefficients of alkene IEs vs HOMO energies and EAs vs LUMO energies. Data are taken from Table S2.

No.	Method	Correlation coefficient (r)		Evaluation of r
		IE vs. HOMO	EA vs. LUMO	
1	STO-3G	-0.492	-0.626	Poor for both
2	3-21G$^{(*)}$	-0.930	-0.882	Excellent for HOMO, near excellent for LUMO
3	6-31G*	-0.947	-0.971	Excellent for both
4	6-31G**	-0.963	-0.968	Excellent for both
5	6-31+G*	-0.961	-0.464	Excellent for HOMO
6	6-311G*	-0.959	-0.901	Excellent for both
7	6-311+G*	-0.960	-0.563	Excellent for HOMO
8	PM3	-0.760	-0.420	Good for HOMO
9	MNDO	-0.827	-0.241	Good for HOMO
10	DFT	-0.873	-0.800	Good for HOMO

Table S2: Alkene IEs, HOMO energies, EAs, and LUMO energies.

No.	Alkene	IE	EA	STO-3G		3-21G[(*)]		6-31G*		6-31G**		6-31+G*		6-311G*		6-311+G*		PM3		MNDO		DFT	
				HOMO[x]	LUMO[x]	HOMO[y]	LUMO[y]	HOMO[y]	LUMO[y]	HOMO[x]	LUMO[x]	HOMO[x]	LUMO[x]	HOMO[x]	LUMO[x]	HOMO[x]	LUMO[x]	HOMO[y]	LUMO[y]	HOMO[aa]	LUMO[aa]	HOMO[z]	LUMO[z]
1		10.52	-1.78	-9.10	8.91	-10.33	5.08	-10.19	5.00	-10.19	4.98	-10.30	2.50	-10.30	4.56	-10.34	2.28	-10.46	1.23	-10.17	1.32	-7.16	0.60
2		9.74	-1.99	-8.61	9.00	-9.83	5.31	-9.72	5.25	-9.71	5.23	-9.81	2.37	-9.82	4.45	-9.84	1.98	-10.10	1.18	-9.97	1.12	-6.66	0.66
3		9.45	-1.73	-8.49	9.07	-9.71	5.08	-9.65	4.99	-9.68	5.16	-9.79	2.15	-9.80	4.17	-9.81	1.88	-10.23	1.19	-9.96	1.15	-6.68	0.66
4	$SiMe_3$	9.80	-1.15	-8.54	8.82	-10.01	4.39	-9.92	4.30	-9.92	4.29	-10.01	1.40	-10.04	3.84			-9.86	0.96	-10.05	1.07	-6.89	0.14
5		9.63	-1.90	-8.56	9.00	-9.79	5.22	-9.70	5.12	-9.70	5.11	-9.79	2.32	-9.81	4.26	-9.83	1.98	-10.15	1.18	-9.94	1.12	-6.63	0.67
6		9.40	-1.78	-8.46	8.95	-9.69	5.04	-9.59	4.92	-9.64	4.99	-9.74	2.12	-9.75	4.08	-9.76	1.85	-10.00	1.20	-9.96	1.11	-6.61	0.66
7	$SiMe_3$	9.00	-1.72	-7.97	9.07	-9.72	5.02	-9.60	4.89	-9.20	4.96	-9.32	1.40	-9.33	3.72			-9.29	0.72	-9.58	1.29	-6.60	0.66
8		9.53	-2.19	-8.52	9.01	-9.77	5.24	-9.70	5.15	-9.69	5.13	-9.79	2.17	-9.81	4.33	-9.82	1.94	-10.26	1.17	-9.96	1.13	-6.71	0.79
9		9.12	-2.22	-8.13	9.04	-9.36	5.25	-9.26	5.18	-9.27	5.47	-9.36	2.29	-9.38	4.25	-9.38	1.92	-9.67	1.11	-9.79	0.93	-6.27	0.83
10		9.12	-2.10	-8.13	9.04	-9.34	5.24	-9.25	5.16	-9.27	5.45	-9.37	2.34	-9.39	4.54	-9.39	1.98	-9.65	1.11	-9.78	0.93	-6.22	0.71
11		8.68	-2.24	-7.78	9.01	-8.93	5.12	-8.86	5.01	-8.97	5.45	-9.06	2.23	-9.08	4.19	-9.07	1.92	-9.40	1.06	-9.63	0.77	-6.14	1.03
12		8.27	-2.27	-7.39	8.91	-8.78	5.46	-8.70	5.36	-8.68	5.37	-8.74	2.29	-8.78	4.13	-8.77	1.92	-9.10	1.02	-9.49	0.62	-5.78	1.07
13	OEt	9.07	-2.24	-7.40	9.19	-9.27	5.52	-9.11	5.51	-9.09	5.49	-9.24	2.38	-9.31	4.36	-9.26	1.98	-9.46	1.33	-9.22	1.30	-5.83	1.16
14	OAc	9.85	-1.19	-9.86	4.47	-9.92	4.39	-9.86	4.47	-9.86	4.47	-10.00	2.06	-9.95	4.22	-10.02	1.87	-9.95	0.58	-10.08	0.50	-6.73	-0.32
15		9.52	-1.92	-8.52	9.00	-9.74	5.08	-9.62	4.98	-9.67	5.16	-9.77	2.28	-9.78	4.31	-9.80	1.96	-10.03	1.17	-9.94	1.12	-6.62	0.68
16		9.48	-1.84	-8.51	9.00	-9.75	5.21	-9.66	5.11	-9.66	5.09	-9.75	2.11	-9.76	4.25	-9.78	1.93	-10.11	1.17	-9.97	1.13	-6.61	0.68
17	F	10.56	-1.91	-10.30	5.14	-10.57	5.01	-10.30	5.15	-10.31	5.11	-10.51	2.31	-10.45	4.58	-10.55	2.08	-10.60	0.71	-10.18	0.67	-7.14	0.55
18	F, F	10.38	-1.84	-8.02	8.42	-10.75	4.84	-10.37	5.16	-10.37	5.13	-10.66	2.24	-10.63	4.70	-10.69	2.08	-10.54	0.21	-10.19	0.02	-7.10	0.40
19	F, F	10.44	-2.18	-8.02	8.43	-10.75	4.89	-10.38	5.26	-10.38	5.23	-10.67	1.99	-10.53	4.25	-10.70	1.80	-10.54	0.23	-10.18	0.04	-7.09	0.49
20	F, F	10.69	-2.39	-8.36	8.49	-10.90	5.07	-10.48	5.50	-10.49	5.48	-10.73	2.14	-10.65	4.72	-10.76	2.00	-10.76	0.23	-10.45	-0.01	-7.29	0.61
21	F, F, F	10.54	-2.45	-7.87	8.19	-11.03	4.87	-10.52	5.52	-10.53	5.50	-10.85	1.97	-10.72	4.62	-10.88	1.87	-10.68	-0.25	-10.46	-0.62	-7.20	0.47
22	F, F, F, F	10.56	-3.00	-7.69	7.95	-11.29	4.82	-10.66	5.81	-10.66	5.81	-11.04	1.89	-10.88	5.62	-11.06	1.88	-10.81	-0.69	-10.74	-1.26	-7.27	0.48
23		8.80	-1.51	-8.01	9.04	-9.10	4.95	-9.11	4.83	-9.11	4.84	-9.40	2.08	-9.42	4.15	-9.42	1.86	-9.80	1.13	-9.78	0.91	-6.49	0.73
24	Cl	10.00	-1.28	-9.09	7.81	-10.26	4.35	-10.14	4.37	-10.14	4.35	-10.22	2.29	-10.25	3.99	-10.26	1.98	-9.84	0.70	-10.39	0.59	-7.15	0.00
25	Cl, Cl	9.91	-0.80	-9.20	6.85	-10.21	3.70	-10.08	3.79	-10.09	3.77	-10.15	2.12	-10.21	3.46	-10.18	1.88	-9.52	0.26	-9.78	0.03	-7.17	0.51
26	Cl, Cl	9.93	-1.11	-9.16	6.90	-10.19	3.80	-10.08	3.92	-10.08	3.90	-10.15	2.04	-10.20	3.63	-10.18	1.77	-9.49	0.29	-10.49	0.38	-7.15	0.39
27	Cl, Cl	10.16	-0.76	-9.37	6.97	-10.35	3.78	-10.23	3.86	-10.23	3.85	-10.29	2.00	-10.34	3.51	-10.32	1.87	-9.74	0.33	-9.84	0.14	-7.30	0.41
28	Cl, Cl, Cl	9.75	-0.59	-9.37	6.14	-10.24	3.26	-10.12	3.43	-10.12	3.42	-10.17	1.82	-10.23	3.16	-10.19	1.70	-9.38	-0.04	-9.69	-0.38	-7.23	-0.78
29	Cl, Cl, Cl, Cl	9.58	-0.30	-9.49	5.47	-10.25	2.82	-10.12	3.05	-10.13	3.05	-10.17	1.75	-10.23	2.84	-10.18	1.71	-9.22	-0.32	-9.61	-0.76	-7.27	-1.07
30	Cl, F	10.21	-1.51	-8.98	7.63	-10.58	4.38	-10.35	4.61	-10.35	4.59	-10.48	2.08	-10.48	4.25	-10.51	1.93	-10.14	0.28	-10.14	0.05	-7.30	0.08
31	Cl, F, Cl	9.80	-1.17	-9.00	6.73	-10.41	3.80	-10.20	4.10	-10.20	4.09	-10.31	1.94	-10.34	3.83	-10.33	1.82	-9.68	-0.10	-9.89	-0.47	-7.23	-0.37
32	Cl, Cl, F, F	9.93	-1.32	-8.72	6.63	-10.60	3.89	-10.28	4.45	-10.28	4.45	-10.45	1.77	-10.43	3.98	-10.47	1.75	-9.76	-0.46	-10.00	-0.96	-7.25	-0.28
33	Cl, F, F, F	10.26	-1.97	-8.29	7.21	-10.88	4.32	-10.44	5.06	-10.44	5.06	-10.69	1.85	-10.62	4.60	-10.71	1.81	-10.18	-0.57	-10.34	-1.10	-7.26	0.08
34		9.24	-2.19	-8.23	9.00	-9.48	5.35	-9.39	5.28	-9.38	5.27	-9.48	2.37	-9.49	4.17	-9.50	1.95	-9.80	1.12	-9.80	0.99	-6.25	0.64
35	$SiCl_3$	11.00	-0.52	-10.44	6.05	-11.29	2.88	-11.21	2.85	-11.17	2.54	-11.18	1.42	-11.26	2.36			-10.59	-1.19	-11.00	-0.20	-8.29	-1.57
36	$Si(OEt)_3$	10.16	-1.11	-8.81	8.58	-9.98	4.57	-10.06	4.30	-10.06	4.29	-10.17	1.64	-10.17	3.94			-9.82	0.88	-10.45	0.79	-6.98	-0.17
37	Cl	10.34	-1.13	-9.26	7.40	-10.46	4.00	-10.35	4.12	-10.35	4.10	-10.44	2.14	-10.47	3.69	-10.47	1.85	-10.31	0.53	-10.48	0.21	-7.24	0.06
38	COOMe	10.74	-0.49	-8.86	6.42	-10.77	3.04	-10.70	3.15	-10.70	3.14	-10.82	2.03	-10.79	2.95	-10.85	1.82	-11.06	-0.11	-10.76	0.16	-7.67	-1.24
39	CN	10.37	-0.17	-8.97	6.52	-10.36	2.94	-10.38	2.91	-10.44	3.10	-10.58	1.82	-10.49	2.65	-10.61	1.62	-10.50	-0.15	-10.43	-0.04	-7.39	-1.27
40	COOMe	10.06	-0.38	-8.62	6.64	-10.22	3.13	-10.15	3.21	-10.19	3.33	-10.31	2.07	-10.29	3.14	-10.32	1.84	-10.52	0.08	-10.46	-0.06	-7.17	-1.13
41	Br	9.90	-1.17	-7.86	8.27	-9.87	4.42	-9.71	4.44	-9.91	4.21	-9.97	1.97	-9.96	3.90	-10.00	1.81	-10.44	-0.06	-10.09	0.53	-6.69	0.19
42	Br	10.18	-0.60	-8.28	7.79	-10.26	4.18	-10.02	3.70	-10.18	3.45	-10.26	1.87	-10.26	3.20	-10.29	1.76	-10.49	-0.21	-10.32	0.10	-6.93	0.16
43	Br	9.58	-1.31	-7.71	8.34	-9.57	4.46	-9.43	4.43	-9.71	4.34	-9.78	2.00	-9.77	4.00	-9.80	1.83	-10.18	-0.07	-9.65	0.41	-6.43	0.26
Correlation coefficient (r)				-0.492	-0.626	-0.930	-0.882	-0.947	-0.971	-0.963	-0.968	-0.961	-0.464	-0.959	-0.901	-0.960	-0.563	-0.760	-0.420	-0.827	-0.241	-0.873	-0.800

Figure S1: Alkene IEs vs HOMO energies calculated by ab initio at HF/3-21G$^{(*)}$ level; data are from Table S2.

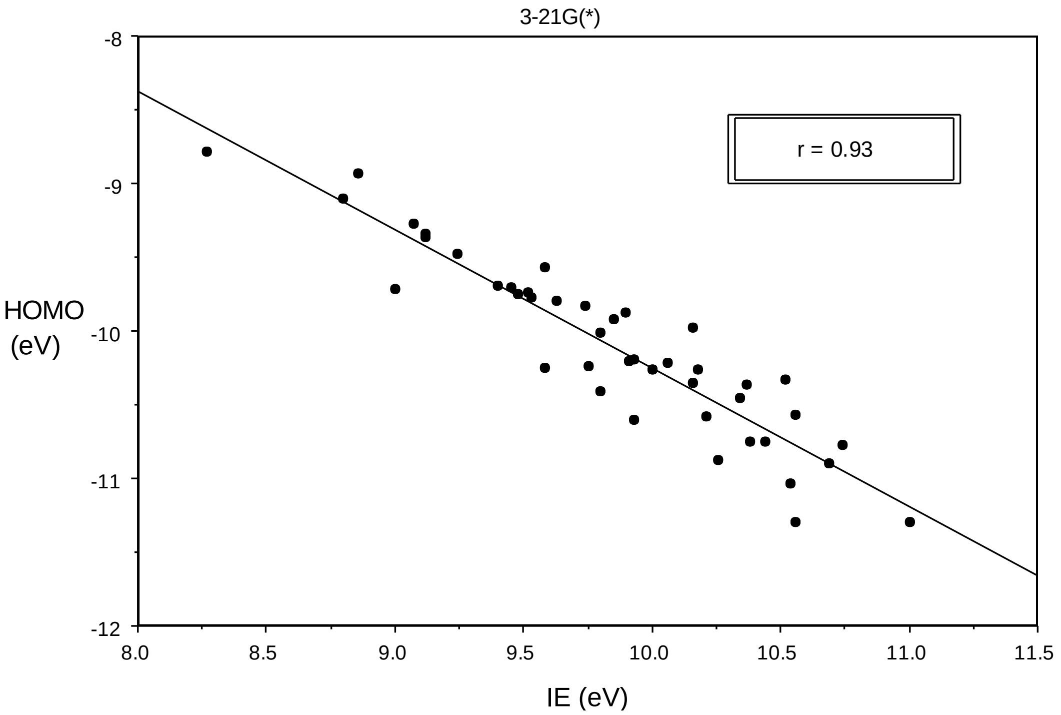

Figure S2: Alkene EAs vs LUMO energies calculated by ab initio at HF/3-21G$^{(*)}$ level; data are from Table S2.

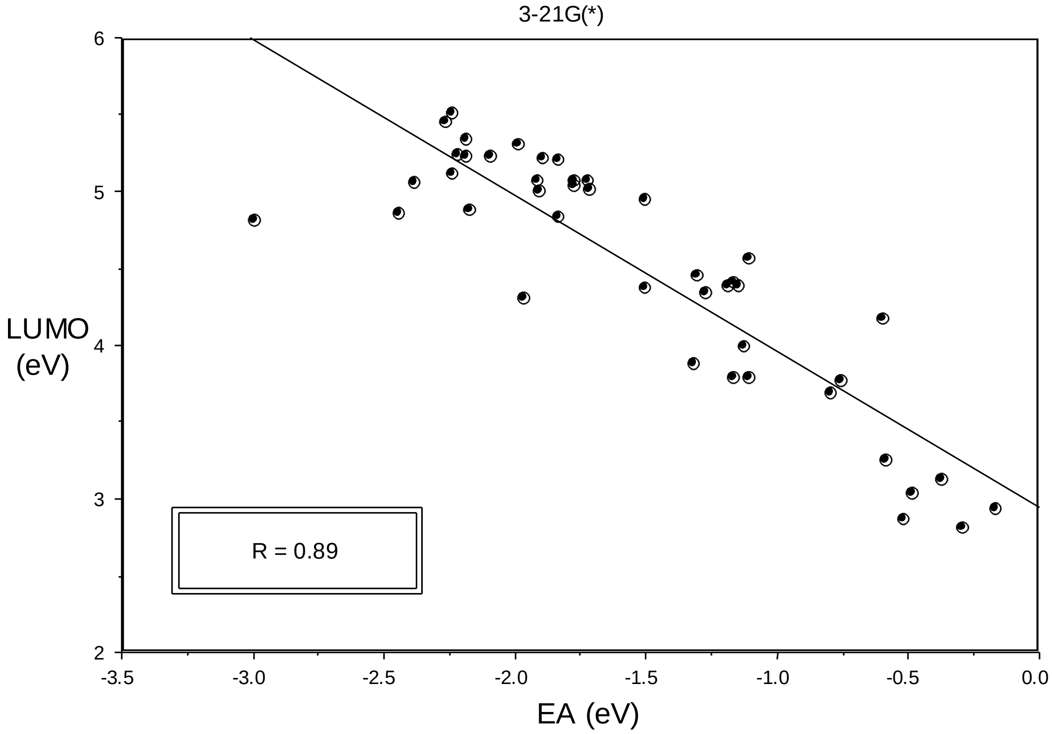

Figure S3: Alkene IEs vs HOMO energies calculated by ab initio at HF/6-31+G* level; data are from Table S2.

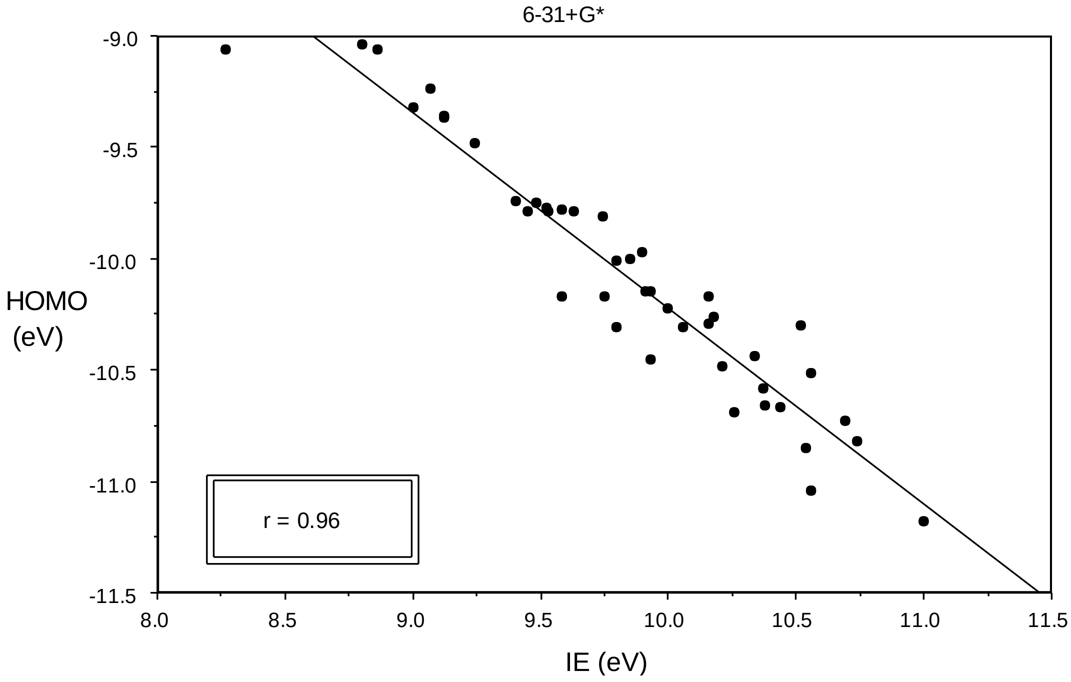

Figure S4: Alkene EAs vs LUMO energies calculated by ab initio at HF/6-31+G* level; data are from Table S2.

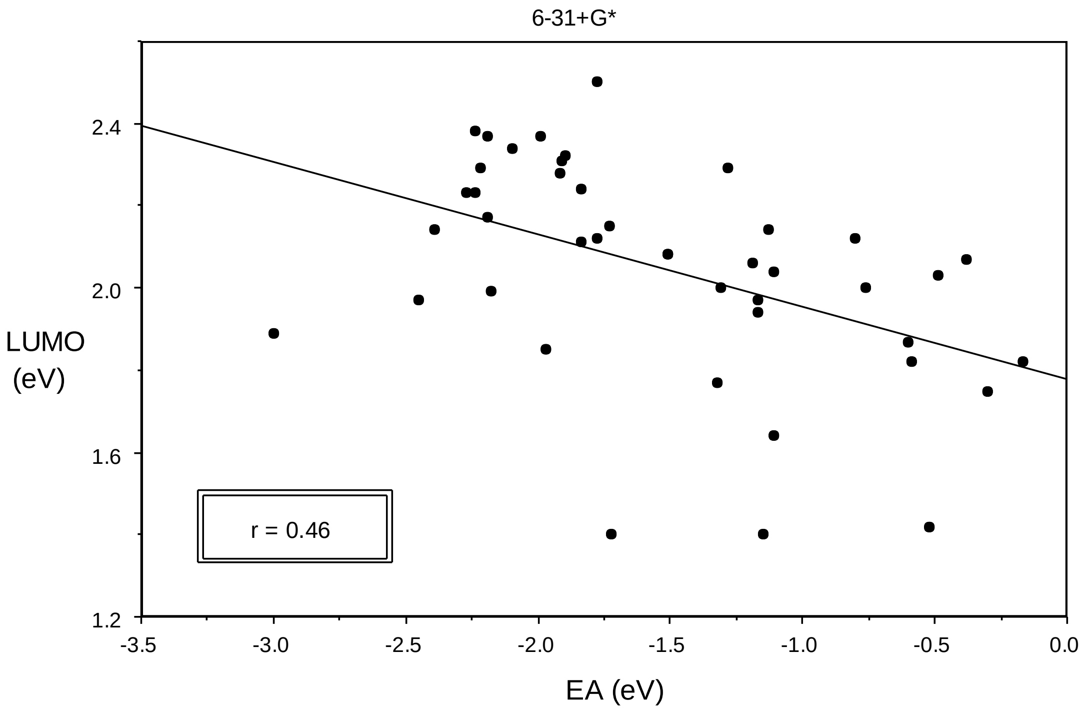

Figure S5: Alkene IEs vs HOMO energies calculated by ab initio at HF/6-311G* level; data are from Table S2.

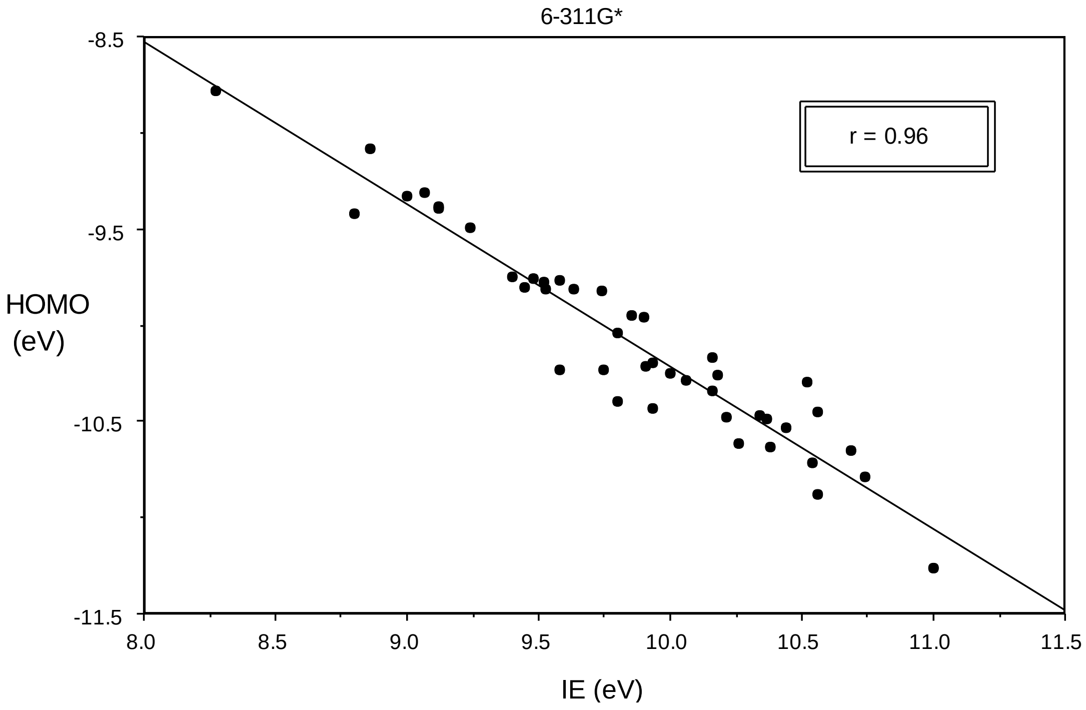

Figure S6: Alkene EAs vs LUMO energies calculated by ab initio at HF/6-311G* level; data are from Table S2.

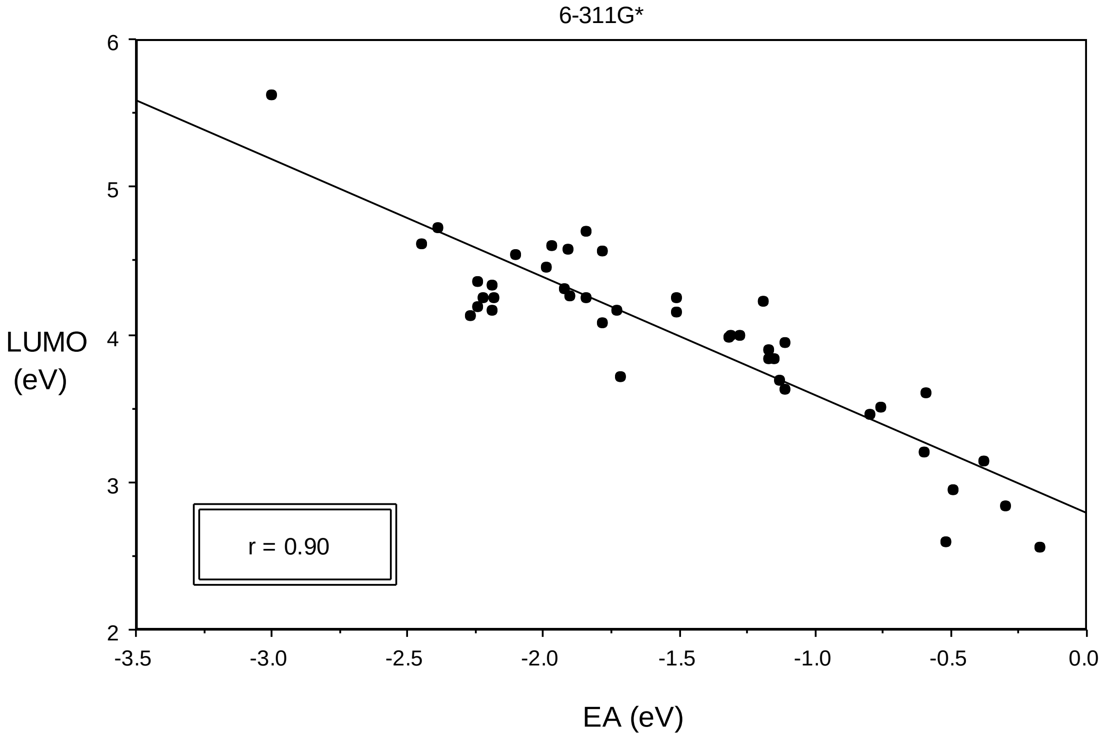

Figure S7: Alkene IEs vs HOMO energies calculated by ab initio at HF/6-311+G* level; data are from Table S2.

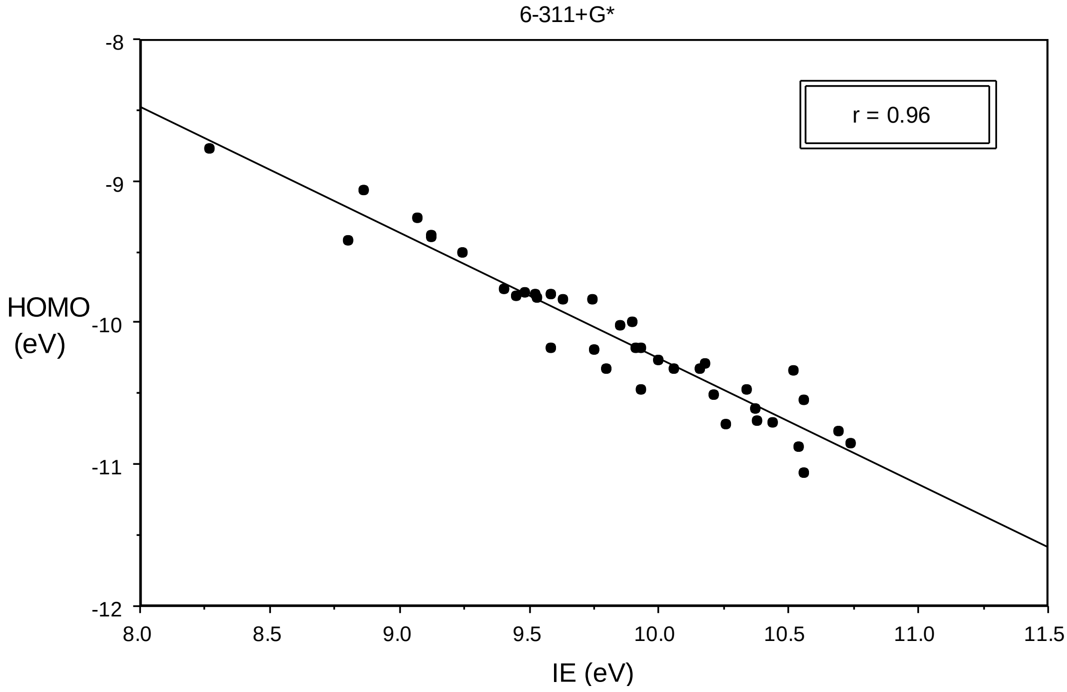

Figure S8: Alkene EAs vs LUMO energies calculated by ab initio at HF/6-311+G* level; data are from Table S2.

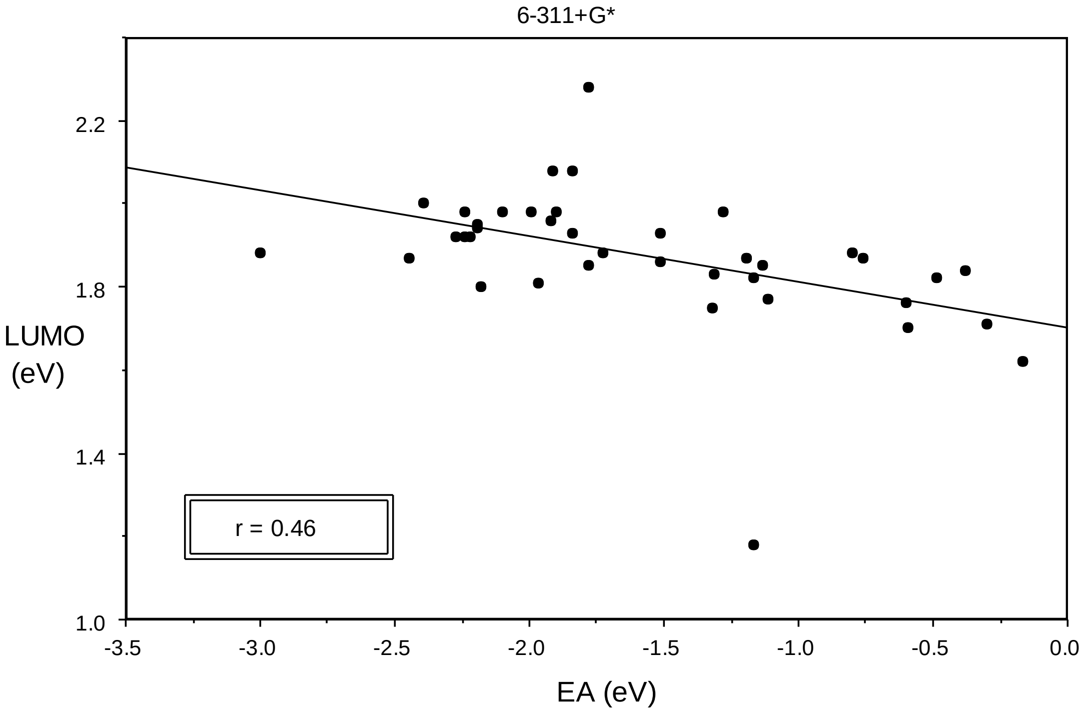

Figure S9: Alkene IEs vs HOMO energies calculated by PM3; data are from Table S2.

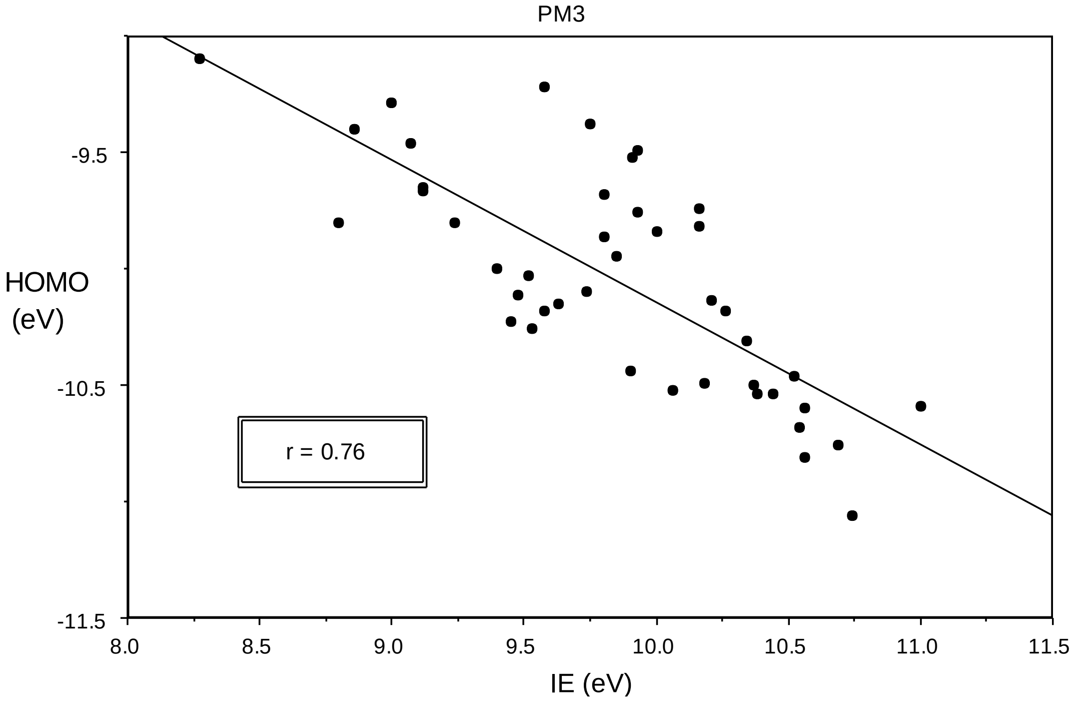

Figure S10: Alkene EAs vs LUMO energies calculated by PM3; data are from Table S2.

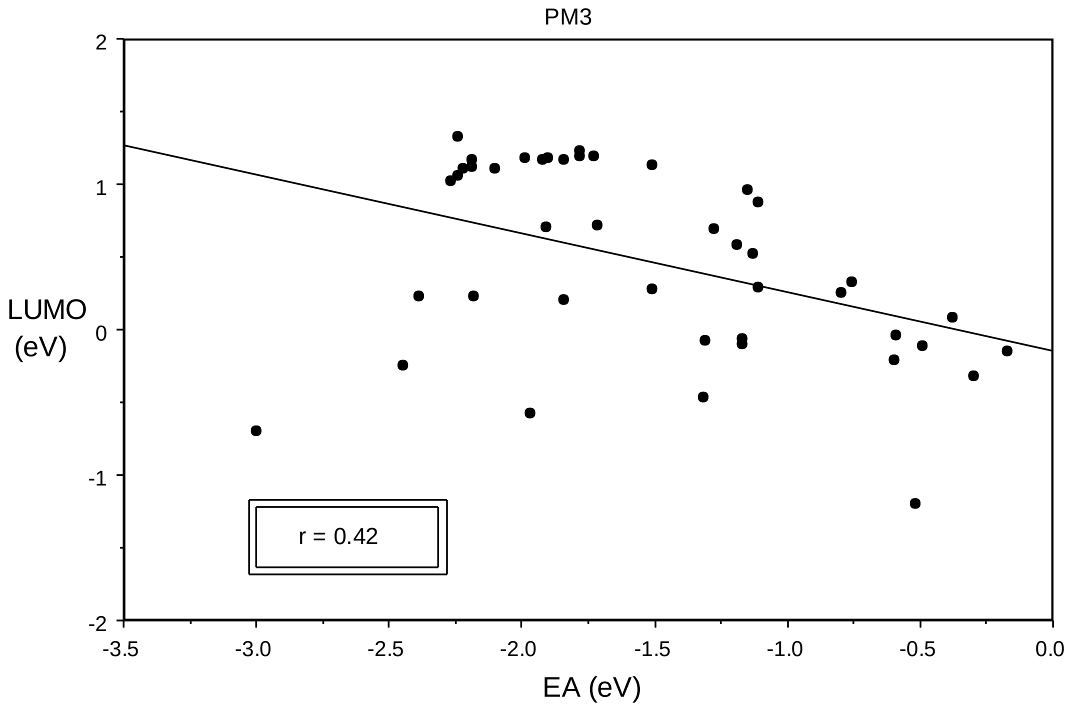

Figure S11: Alkene IEs vs HOMO energies calculated MNDO; data are from Table S2.

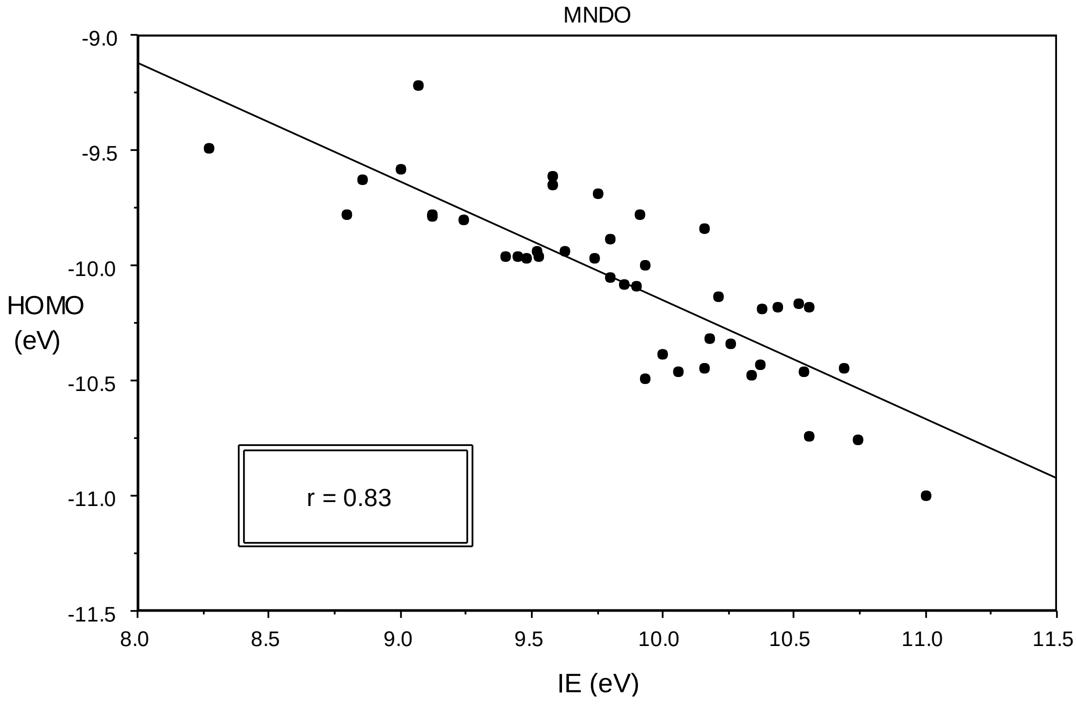

Figure S12: Alkene EAs vs LUMO energies calculated by MNDO; data are from Table S2.

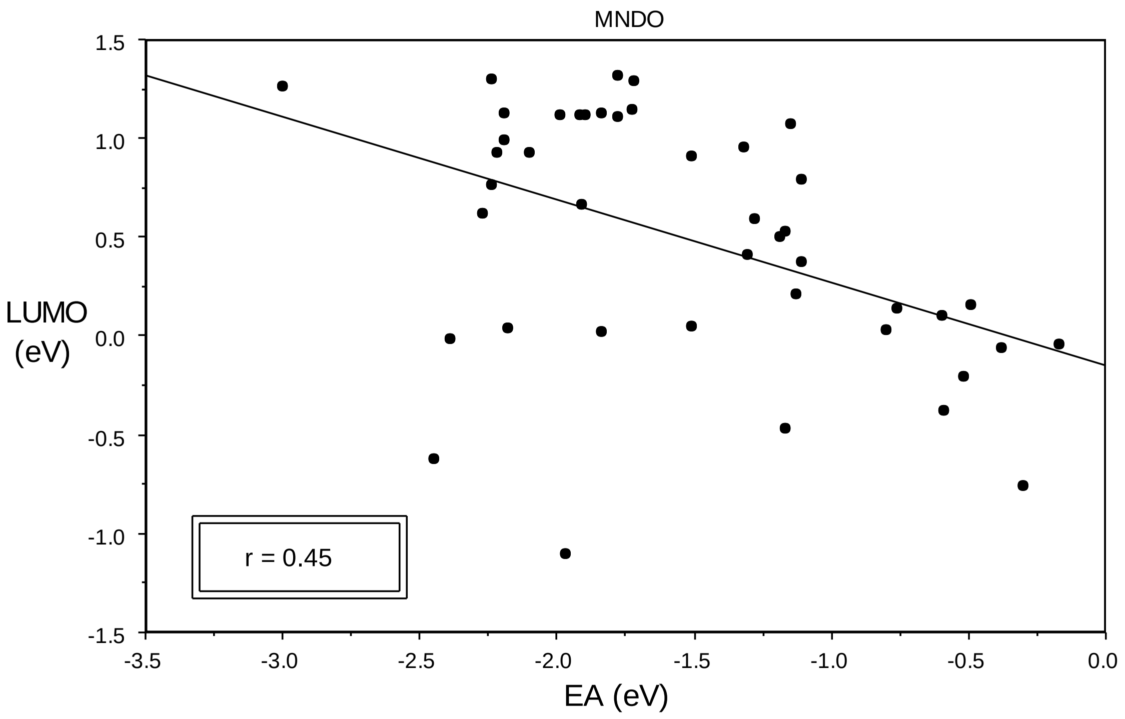

Figure S13: Alkene IEs vs HOMO energies calculated DFT (B3LYP/6-31G*); data are from Table S2.

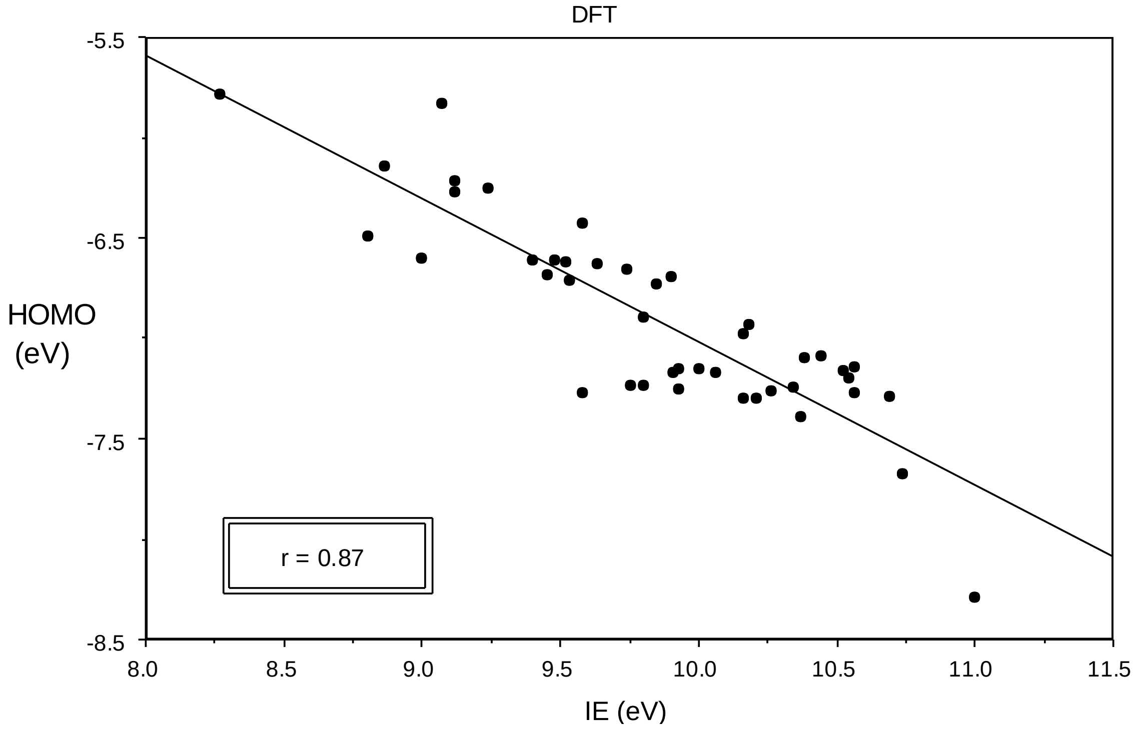

Figure S14: Alkene EAs vs LUMO energies calculated by DFT (B3LYP/6-31G*); data are from Table S2.

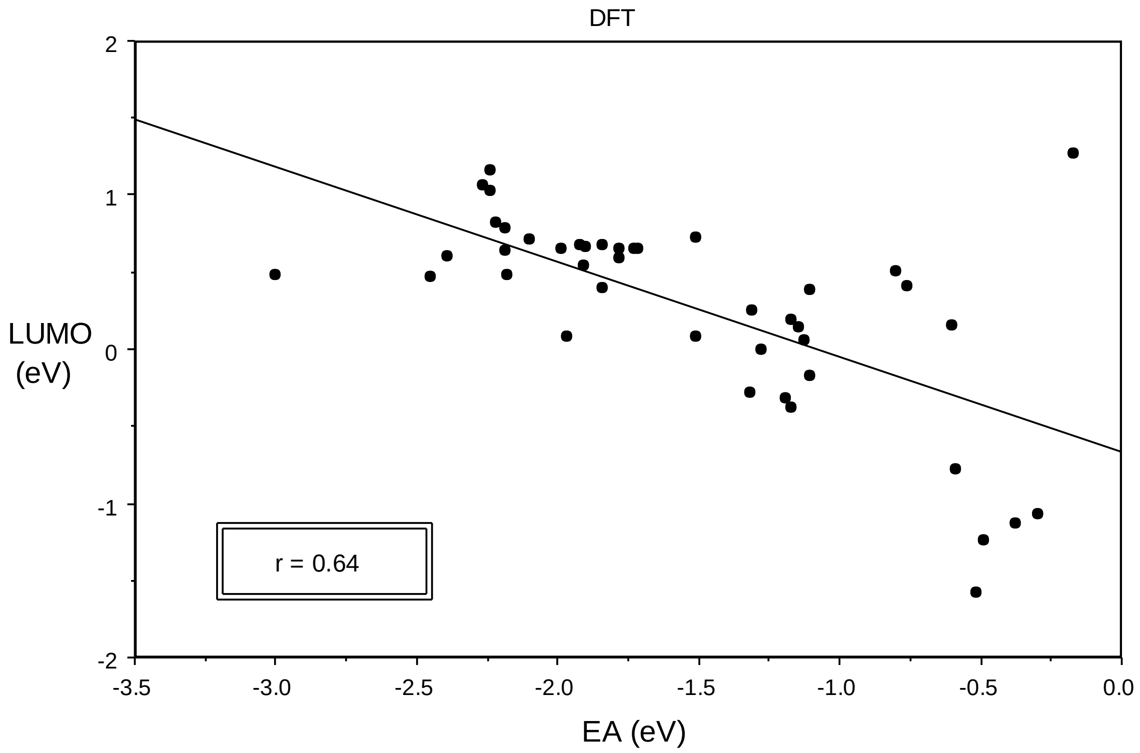
DFT
LUMO (eV)
EA (eV)
r = 0.64
2
1
0
-1
-2
-3.5
-3.0
-2.5
-2.0
-1.5
-1.0
-0.5
0.0
